# A Case of Onychomycosis Caused by *Rhodotorula glutinis*

**DOI:** 10.1155/2014/563261

**Published:** 2014-09-25

**Authors:** Hatice Uludag Altun, Tuba Meral, Emel Turk Aribas, Canan Gorpelioglu, Nilgun Karabicak

**Affiliations:** ^1^Department of Clinical Microbiology, Faculty of Medicine, Turgut Ozal University, Emek, 06510 Ankara, Turkey; ^2^Department of Dermatology, Faculty of Medicine, Turgut Ozal University, Emek, 06510 Ankara, Turkey; ^3^Mycology Reference Laboratory, Public Health Institution of Turkey, Sıhhıye, 06100 Ankara, Turkey

## Abstract

*Rhodotorula* spp. have emerged as opportunistic pathogens, particularly in immunocompromised patients. The current study reports a case of onychomycosis caused by *Rhodotorula glutinis* in a 74-year-old immunocompetent female. The causative agent was identified as *R. glutinis* based on the pinkish-orange color; mucoid-appearing yeast colonies on Sabouraud Dextrose Agar at 25°C; morphological evaluation in the Corn Meal-Tween 80 agar; observed oval/round budding yeast at 25°C for 72 hours; no observed pseudohyphae; positive urease activity at 25°C for 4 days; and assimilation features detected by API ID 32C kit and automated Vitek Yeast Biochemical Card 2 system. Antifungal susceptibility test results were as follows: amphotericin B (MIC = 0.5 *µ*g/mL), fluconazole (MIC = 128 *µ*g/mL), itraconazole (MIC = 0.125 *µ*g/mL), voriconazole (MIC = 1 *µ*g/mL), posaconazole (MIC = 0.5 *µ*g/mL), anidulafungin (MIC = 0.5 *µ*g/mL), and caspofungin (MIC = 16 *µ*g/mL). Antifungal therapy was initiated with oral itraconazole at a dose of 400 mg/day; seven-day pulse therapy was planned at intervals of three weeks. Clinical recovery was observed in the clinical evaluation of the patient before the start of the third cure. Although *R. glutinis* has rarely been reported as the causative agent of onychomycosis, it should be considered.

## 1. Introduction

Onychomycosis is the general name for a mycotic nail infection caused by dermatophytes, yeasts, and nondermatophyte molds. The prevalence of onychomycosis has been reported to be 2–30% and has increased in recent years [1]. Old age, toenail deformities, onychodystrophy, diabetes mellitus, psoriasis vulgaris, and psoriasis unguium, cellular immunity disorders, genetic predisposition, peripheral arterial circulatory disorder, other circulatory disorders, nail and nail fold microtrauma, heavy perspiration/hyperhidrosis pedum, and immunosuppression (HIV/AIDS) should be considered as risk factors for onychomycosis [2, 3].

Onychomycosis, which constitutes 50% of all nail diseases, is observed with clinical findings like onycholysis, subungual hyperkeratosis, discoloration, crumbly thick nails, or white patches on the nail surface [4]. Fungi that cause onychomycosis are categorized into three groups: dermatophytes, yeasts, and nondermatophyte molds [5]. Dermatophytes that cause onychomycosis according to asexual reproduction feature three groups (*Trichophyton*,* Epidermophyton*, and* Microsporum*) the most frequently observed species of which are* Trichophyton* and* Epidermophyton* [6]. The most common agents of onychomycosis among yeasts are* Candida albicans* and* Candida parapsilosis* [2, 7]. Onychomycosis, according to the state of the agent to penetrate the nail, can be classified into one of five types, which are distal-lateral subungual onychomycosis (DLSO), proximal subungual onychomycosis (PSO), superficial white onychomycosis (SWO), candidal onychomycosis (CO), and total dystrophic onychomycosis (TDO) [8]. The most common clinical form is DLSO [9]. Toenails are more frequently involved DLSO and* T. rubrum* is the most common pathogen [10].

*Rhodotorula* spp. are uncommon among the agents of onychomycosis in the literature. To date two cases have been reported as the causative agents of onychomycosis (*R. minuta* and* R. mucilaginosa*) [11, 12].

## 2. Case Report

**Figure 1:**
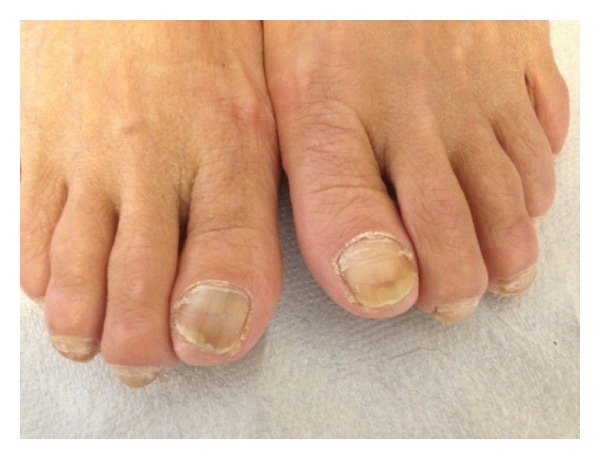
Discoloration and onycholysis image in bilateral toenail.

A 74-year-old woman was admitted to the dermatology outpatient clinic of our hospital with complaints of deformity and thickening of the toenails that had continued for nearly three months. In the dermatological examination, of bilateral toenails, subungual hyperkeratosis in varying degrees, yellow-brown discoloration, and onycholysis were observed (Figure 1). The general physical examination was normal. Chronic diseases were absent, with the exception of hypertension. The patient did not have chronic or familial genetic diseases that could have caused nail disorders, malignancy, or previous trauma. The patient revealed that she had traveled to the Far East within the previous six months. The patient reported no use of systemic corticosteroid or broad-spectrum antibiotics. Other immunosuppressive conditions associated with* Rhodotorula* infection, such as AIDS, were absent. The patient's toenail samples were sent to the microbiology laboratory for fungal culture.

Clinically suspected of onychomycosis, according to nail culture results, the patient was diagnosed with DLSO caused by* R. glutinis*.

Antifungal therapy was initiated with 400 mg/day oral itraconazole; seven-day pulse therapy was planned at intervals of three weeks. The clinical evaluation of the patient before the start of the third cure, clinical recovery was detected. The patient's treatment is still underway; a fungal culture was planned again after six treatments.

### 2.1. Fungal Identification

**Figure 2:**
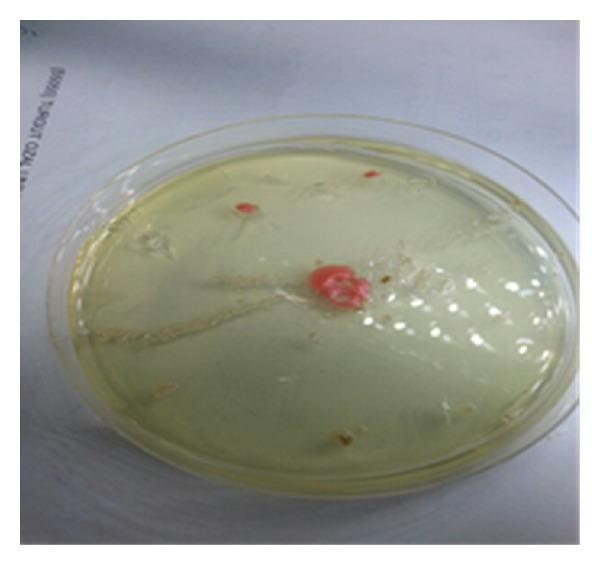
The yeast colonies on SDA at the first cultivation of the nail samples.

**Figure 3:**
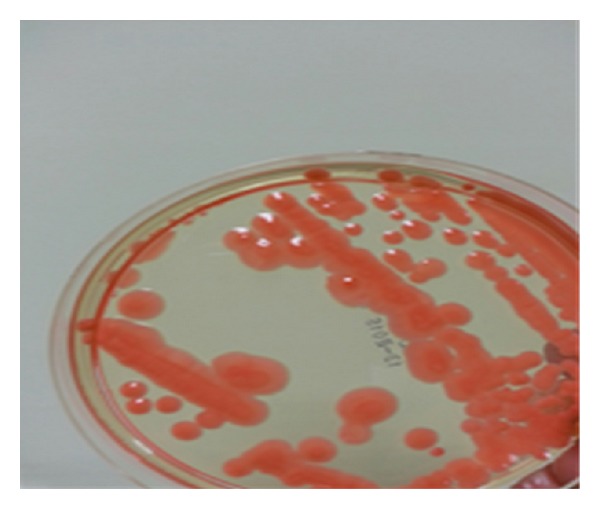
The yeast colonies on the SDA after subcultivation.

The toenail samples were cultured on 2 Sabouraud Dextrose Agars (SDA; Salubris, Turkey) in the microbiology laboratory. One of SDA was incubated at 37°C and the other was incubated at 25°C. SDA, which was incubated at room temperature for four days, was observed to be a pinkish-orange pigmented colony (Figure 2). The pure passage of growing colonies was performed on the SDA medium (Figure 3). The Gram staining of these colonies was observed in the yeast cells forming blastospores. The yeast was thought to be* Rhodotorula*, due to its orange-pink pigmented appearance. The urease test was performed. The two strains of* C. albicans* (American Type Culture Collection (ATCC) 10231 and ATCC 24433) were used as a negative control, and* Cryptococcus neoformans* (ATCC 24067) was used as the positive control. Urease activity was positive.

The tested pathogen was indicated as* Rhodotorula glutinis/mucilaginosa* according to the Vitek automated identification system (bioMérieux, France) using Yeast Biochemical Card 2 (YCB). The species identification of the strains was performed at the Public Health Institution of Turkey-Mycology Reference Laboratory (PHIT-MRL). The pinkish-orange color, mucoid-appearing yeast colonies on SDA at 25°C, the morphological evaluation in the Corn Meal-Tween 80 agar, observed oval/round budding yeast at 25°C for 72 hours, no pseudohyphae, determination of positive urease activity at 25°C for four days, assimilation features detected by API ID 32C (bioMérieux, France) kit, evaluated together with conventional mycological methods identified the species as* Rhodotorula glutinis* [13].

### 2.2. In Vitro Susceptibility Test

Susceptibility tests of the strain to amphotericin B, fluconazole, itraconazole, voriconazole, posaconazole, anidulafungin, and caspofungin were performed using the microdilution method (M27-A3), recommended CLSI in PHIT-MRL. Quality control (QC) was performed using* Candida parapsilosis* ATCC 22019 and* Candida krusei* ATCC 6258. Due to the fact that after 24 hours of incubation there was no bacterial growth in growth control well and poor growth after 48 hours, minimum inhibitory concentration (MIC) was determined, after a 72-hour incubation according to the CLSI-M27A3 recommended resistance limit values [14]. Antifungal susceptibility test results were as follows: amphotericin B (MIC = 0.5 *μ*g/mL), fluconazole (MIC = 128 *μ*g/mL), itraconazole (MIC = 0.125 *μ*g/mL), voriconazole (MIC = 1 *μ*g/mL), posaconazole (MIC = 0.5 *μ*g/mL), anidulafungin (MIC = 0.5 *μ*g/mL), and caspofungin (MIC = 16 *μ*g/mL).

## 3. Discussion

*Rhodotorula* spp. are yeasts that are prevalent in nature. The* Rhodotorula* species are particularly found in soils, lakes, milk, fruit juices, and the resident flora of moist skin in humans.* Rhodotorula* infections are more frequently isolated in the Asia-Pacific region [15].

Infections that are caused by the* Rhodotorula* species are rare.* Rhodotorula* spp. are accepted as pathogen in recent years. Recently, catheter infections caused by* Rhodotorula* spp. are seen more frequently because of invasive procedures and, in particular, the increased use of central venous catheter [16]*. Rhodotorula mucilaginosa*,* R. glutinis*, and* R. minuta* are the species that cause disease in humans [16, 17].* Rhodotorula* spp. were found to be the fourth most frequently observed species among non-*Candida* yeasts isolated from clinical specimens. The fact that invasive infections are reported less frequently in epidemiological studies should be taken into consideration [18].

*Rhodotorula* spp. are identified by the growth of the agent on cultures. Many morphological and physiological characteristics of the* Rhodotorula* species are similar to the* Cryptococcus* species in identification. Both types exhibit round-shaped, encapsulated yeast cells and urease activity, and fermenting carbohydrates specifications are determined to be positive.* Rhodotorula* species from* Cryptococcus* are separated by evident carotenoid pigments and not assimilating inositol. If there are visible capsules, they are typically thin different from* C. neoformans* [19].

The incidence of* Rhodotorula* spp. is 0.02% among fungal infections in patients with hematological malignancies [20]. Central venous catheters in patients with* Rhodotorula* fungemia are significant as both risk factor and prognostic factor [21, 22]. Another major risk factor is severe neutropenia. Steroid administration and the use of broad-spectrum antibiotics are also risk factors [15]. The cases of* Rhodotorula* infection reported in literature included fungemia, meningitis, endocarditis, skin lesions, eye infections, onychomycosis, and peritonitis [11, 12, 17, 23].* Rhodotorula mucilaginosa* was the most common species of* Rhodotorula* fungemia, followed by* Rhodotorula glutinis* [17]. According to a recent document,* R. glutinis* could be present in the skin of early systemic sclerosis patients at higher levels than in normal skin, raising the possibility that it could be triggering the inflammatory response found in systemic sclerosis [24].

*Rhodotorula* infections in immunocompetent patients are extremely rare. In the literature,* Rhodotorula* spp. were reported as a factor of onychomycosis in two cases.* Rhodotorula mucilaginosa* and* R. minuta* were found as the causative agent in those cases [11, 12]. One other case reported in the literature is nail psoriasis, masqueraded by secondary infection with* R. mucilaginosa* [25]. In these three cases, the patients were immunocompetent, as in the current case [11, 12, 25].

Treatment approaches against infections due to* Rhodotorula* are still controversial. In vitro susceptibility tests detected that amphotericin B, itraconazole, voriconazole, and 5-flucytosine are the most active antifungal agents, although voriconazole, particularly against* R. mucilaginosa* isolates, did not exhibit adequate activity [26]. In the literature, the low MICs to both posaconazole and ravuconazole were reported, though there is not sufficient clinical experience [27]. On the other hand, resistance to fluconazole, caspofungin, and micafungin was observed [21, 26, 28]. The mechanism of resistance to fluconazole is uncertain, but reported higher MIC values may indicate intrinsic resistance [12]. In the current case, according to the results of antifungal susceptibility tests, the MIC value for fluconazole was 128 *μ*g/mL. High MIC values for fluconazole in the literature in patients with* Rhodotorula* onychomycosis (≥128 = 16 *μ*g/mL) were similar to the current results [11, 12]. MIC values determined for voriconazole (1 *μ*g/mL) and posaconazole (0.5 *μ*g/mL) were higher than those detected for itraconazole (0.125 *μ*g/mL). Higher MIC values for caspofungin determined were similar to articles in the literature; the MIC value of anidulafungin was 0.5 *μ*g/mL [21, 26, 28].

In the current case, according to the patient's clinical symptoms and the results of the antifungal susceptibility test, the patient was administered itraconazole therapy due to the sensitive results for this antifungal agent (itraconazole MIC = 0.125 *μ*g/mL), which is effective on onychomycosis caused by* R. mucilaginosa*. In the literature, in one case of onychomycosis caused by* R. minuta*, following the administration of itraconazole treatment (itraconazole MIC value <0.125 *μ*g/mL), it was reported that the patient fully recovered [12].

In conclusion,* Rhodotorula* spp. are rarely seen yeasts that can cause infection especially in immunosuppressed people. In the literature,* R. glutinis* is rarely reported as the causative agent of onychomycosis, although it should be considered as such.
